# Multimodal Radiomics Model Combining HR‐VWI and Clinical Features for Identifying Symptomatic Basilar Atherosclerotic Plaques

**DOI:** 10.1155/bmri/7319324

**Published:** 2026-06-02

**Authors:** Juan Bai, Caixian Yang, Ning Li, Xiaoyong Hao, Lina Zhu, Jingxi Yan, Jiang Wu, Mengzhu Wang

**Affiliations:** ^1^ Magnetic Resonance Room, Shanxi Cardiovascular Hospital, Shanxi Key Laboratory of Heart Failure Precision Medicine, Cardiovascular Hospital Affiliated to Shanxi Medical University, Taiyuan, China, sxmu.edu.cn; ^2^ Department of Radiology, Shanxi Provincial People′s Hospital, Taiyuan, China; ^3^ MR Research Collaboration Team, Siemens Healthineers Ltd., Beijing, China

**Keywords:** atherosclerosis, basilar artery, high-resolution vessel wall imaging, plaque, radiomics

## Abstract

**Objective:**

This study is aimed at exploring the value of a high‐resolution vessel wall imaging (HR‐VWI)–based radiomics approach for identifying symptomatic basilar atherosclerotic plaques.

**Materials and Methods:**

This retrospective study included 154 patients, hospitalized at Shanxi Cardiovascular Hospital for cerebrovascular disease, who had basilar artery stenosis and underwent HR‐VWI examinations between July 2020 and December 2023. The patients were randomly divided into training and validation sets in a 7:3 ratio and further classified into symptomatic and asymptomatic groups based on the presence or absence of acute infarction lesions in the basilar artery supply area, respectively. Clinical and traditional imaging features were collected for all patients. Manual plaque segmentation and radiomics feature extraction were performed on the layers of basilar artery plaques identified in the plain‐scanning and enhanced HR‐VWI images. Feature denoising was performed using minimum redundancy maximum correlation, and the least absolute shrinkage and selection operator (LASSO) algorithm was applied to reduce feature variables. The most suitable features were selected through univariate and multivariate logistic regression analyses. The plain‐scanning, enhanced, and plain − scanning + enhanced models were established. Finally, a combined model was constructed by integrating selected clinical and traditional imaging features (intraplaque hemorrhage [IPH], enhanced plaque signal) with the plain − scanning + enhanced HR − VWI radiomics features. The recognition efficiency of each model for symptomatic basilar atherosclerotic plaques was evaluated using the receiver operating characteristic (ROC) curve and the area under the receiver operating characteristic curve (AUC).

**Results:**

A statistically significant difference in age was observed between symptomatic and asymptomatic groups (*p* < 0.05). Conventional imaging features, including IPH, vessel diameter at stenosis, lumen area at stenosis, enhanced plaque signal, stenosis rate, and plaque load, also demonstrated statistically significant differences between the two groups (*p* < 0.05). Firth‐penalized logistic regression analysis identified IPH and enhanced plaque signal as risk factors for symptomatic basilar artery plaques. In the training set, the AUC values for detecting symptomatic plaques were 0.813, 0.853, and 0.898 for plain‐scanning, enhanced, and plain − scanning + enhanced models, respectively. In the validation set, the AUC values were 0.786, 0.848, and 0.880, respectively. The efficacy of models was in the order of plain − scanning + enhanced model > enhanced model > plain − scanning model. The combined model, integrating IPH and enhanced plaque signal with the plain − scanning + enhanced HR − VWI radiomics features, achieved an AUC of 0.916 in the training set, with sensitivity, specificity, and accuracy of 0.821, 0.941, and 0.879, respectively, for predicting symptomatic basilar artery plaques. In the validation set, the AUC value was 0.891, with sensitivity, specificity, and accuracy of 0.792, 0.870, and 0.830, respectively. The combined model demonstrated superior performance compared with the plain − scanning + enhanced model.

**Conclusion:**

Our HR‐VWI–based radiomics models can accurately distinguish symptomatic from asymptomatic basilar atherosclerotic plaques. It is superior to the traditional model in the identification of high‐risk plaques.

## 1. Introduction

With an incidence of 18 cases per 100,000 person/year [1], posterior circulation ischemic stroke is a cerebral infarction occurring in the brain region supplied by the vertebrobasilar artery, accounting for 20%–25% of all ischemic strokes [2–5]. It has a mortality at 1 month of 3.6%–11% and a disability at 3 months of 6.9%–19.8% [1]. The basilar artery, being the main blood vessel in the posterior circulation of the brain, plays a key role in posterior circulation ischemic stroke.

Studies have demonstrated that the incidence of ischemic stroke strongly correlates with arterial stenosis, influencing clinical intervention strategies. However, relying solely on the stenosis degree to stratify stroke risk is inadequate [6]. The likelihood of cerebrovascular events in patients with intracranial atherosclerosis depends largely on atherosclerotic plaque stability. Unstable plaques are also known as vulnerable plaques. Pathological features include (1) intraplaque hemorrhage (IPH); (2) lipid‐rich necrotic core (LRNC), thin fiber cap; (3) fibrous cap rupture (FCR); (4) juxtalumenal calcium nodule; (5) inflammation; and (6) neovasculature [7]. Due to their instability, vulnerable plaques may progressively worsen vascular stenosis or rupture over time, thereby triggering symptoms and transforming into symptomatic plaques.

At present, the imaging techniques for detecting head and neck arterial plaques mainly include ultrasound, CT angiography (CTA), digital subtraction angiography (DSA), high‐resolution vessel wall imaging (HR‐VWI), and so on. Conventional carotid ultrasound is sensitive to the identification of carotid plaques and changes in blood flow dynamics but cannot observe the relevant conditions of intracranial arteries. Head and neck CTA can effectively identify plaques, including the degree of luminal stenosis and analysis of calcified components. However, its evaluation of nonnarrowed plaques or low‐density plaques is not accurate enough, and its diagnostic effect on vulnerable plaques is limited. DSA can accurately display the degree and extent of vascular stenosis, making it the “gold standard” for diagnosing vascular diseases. As HR‐VWI has advanced, researchers are now focusing more on plaque stability. HR‐VWI has considerably progressed in carotid plaque research, effectively displaying plaques and detecting intraplaque components such as lipids, fibrous caps, and hemorrhage. HR‐VWI can reveal the presence of plaque, detect any bleeding within the plaque, and assess plaque stability based on the degree of enhancement due to the small size of intracranial artery plaques. However, HR‐VWI is complex despite its reliability and noninvasiveness and requires expertise in evaluating various plaque components, rendering it an inherently subjective qualitative task.

Radiomics has exhibited promising performance in tumor diagnosis, staging, treatment evaluation, and outcome prediction [8, 9]. However, its application in cardiovascular and cerebrovascular diseases has not been explored much.

This study is aimed at developing a model integrating clinical and traditional imaging features based on HR‐VWI and evaluate its effectiveness in identifying symptomatic basilar atherosclerotic plaques, providing a reference for future risk assessment of basilar atherosclerotic plaques.

## 2. Materials and Methods

### 2.1. Study Population

This retrospective study included patients hospitalized at Shanxi Cardiovascular Hospital for cerebrovascular disease between July 2020 and December 2023.

The inclusion criteria were as follows: (1) age > 18 years, (2) magnetic resonance angiography (MRA) evidence of basilar artery stenosis, and (3) patients exhibiting localized vessel wall thickening at the site of basilar artery stenosis on HR‐VWI.

The exclusion criteria were as follows: (1) poor image quality with considerable motion artifacts preventing analysis; (2) evidence of cardiogenic stroke [10]; (3) bilateral transient ischemic attack (TIA)/stroke, undetermined cerebral hemisphere involvement, or inability to accurately identify the responsible plaque; (4) primary intracranial diseases or nonatherosclerotic vascular conditions (e.g., vasculitis and arterial entrapment); (5) arterial stenosis caused by radiation therapy; and (6) contraindications to MRI (e.g., pacemaker and severe claustrophobia) or gadolinium use. Patients meeting any exclusion criterion were excluded.

Initially, 180 patients were included, among whom 26 were excluded due to poor image quality secondary to motion artifacts and other related issues. Ultimately, a total of 154 patients were included in this study, consisting of 117 males and 37 females, aged 34–82 years (mean age: 61.44 ± 8.697 years). The participants were divided into training and validation sets at a ratio of 7:3. According to the presence or absence of acute infarction in the basilar artery territory on diffusion‐weighted imaging, participants were classified into symptomatic and asymptomatic groups, respectively. To eliminate confounding effects, a comprehensive vascular evaluation was performed to exclude symptoms resulting from other posterior circulation arteries, including the vertebral artery and posterior cerebral artery. The study was approved by the Medical Ethics Committee of Shanxi Cardiovascular Hospital (Ethical Number 2023xxg003).

### 2.2. Instruments and Methods

A 3‐T MR scanner (MAGNETOM Skyra; Siemens Healthineers, Forchheim, Germany) with a 32‐channel high‐resolution head and neck coil was used. The patients were positioned supine, and images were acquired using a head‐first approach.

The scanning sequences and parameters used in this study were as follows: (a) 3D time‐of‐flight MRA: repetition time (TR) = 20.0 ms, echo time (TE) = 3.50 ms, field of view (FOV) = 220 × 196  mm^2^, acquisition matrix = 320 × 240, slice thickness = 0.60 mm, flip angle = 20°, 176 slices total, and acquisition time = 4 min and 58 s. (b) 3D T1‐weighted imaging using sampling perfection with application‐optimized contrasts at varying flip angles (3D T1WI SPACE): TR = 900 ms, TE = 15.0 ms, slice thickness = 0.63 mm, FOV = 200 × 200  mm^2^, acquisition matrix = 320 × 320, 224 slices per slab, and acquisition time = 7 min and 36 s. (c) Enhanced 3D T1WI SPACE: Contrast‐enhanced imaging was performed after intravenous bolus administration of gadobutrol injection (Bayer AG, Germany) at a dose of 0.1 mmol/kg (0.1 mL/kg) through antecubital venous access, followed by a 20‐mL saline flush at 2.0 mL/s. The scanning parameters were identical to those of nonenhanced 3D T1WI SPACE to ensure quantitative comparability.

### 2.3. Image Analysis

#### 2.3.1. Plaque Identification

First, the site of basilar artery stenosis was identified on the MRA image, and the stenotic vessel was reconstructed using the 3D HR‐VWI image. The eccentric thickening of the vessel wall was observed at the stenotic site, perpendicular to the vessel′s diameter, and the plaque causing the most severe stenosis was selected as the target plaque.

#### 2.3.2. Analysis of Traditional Imaging Features of Plaques

The HR‐VWI and MRA images were imported into the postprocessing workstation (syngo.via; Siemens Healthineers). IPH is characterized by high signal intensity on T1WI (higher than 150% of the signal intensity of reference muscle tissue). The following parameters were measured and calculated: plaque diameter at stenosis (*D*_p_) (defined as the longest diameter measured at the largest level of the plaque), vessel diameter at stenosis (*D*_v_) (defined as the maximum distance between the outer walls of the vessel), normal lumen diameter at the proximal end of the stenosis, stenotic lumen area (*S*_L_) (defined as the area of the lumen at the largest level of the plaque, measured along the inner wall of the vessel), vessel area at stenosis (*S*_V_) (defined as the area of the vessel at the stenosis, outlined along the outer wall of the vessel), plain‐scanning plaque signal and plain‐scanning peripheral gray matter signal, enhanced plaque signal and enhanced peripheral gray matter signal, distal normal vessel area, narrowing rate, plaque burden, enhancement rate, and reshaping index.

Narrowing rate=1−Dv/normal lumen diameter proximal to stenosisPlaque burden=1−SL/SVEnhancement rate=enhanced plaque signal/enhanced peripheral gray matter signalplain−scanning plaque signal/plain−scanning peripheral gray matter signal−1Reshaping index=SVdistal normal vessel area

### 2.4. Collection of Clinical Features

Data collected on clinical risk factors for cardiovascular disease included sex, age, history of hypertension, diabetes mellitus, hyperlipidemia, smoking, and alcohol consumption.

### 2.5. Construction of Radiomics Models

#### 2.5.1. Outlining Regions of Interest (ROIs)

**Figure 1:**
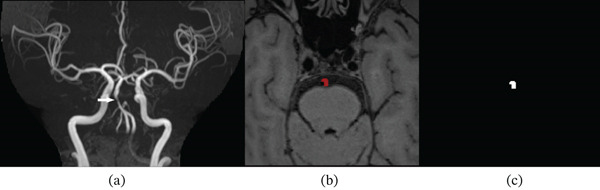
Procedure of radiomics segmentation: (a) TOF demonstrated basilar artery stenosis. (b) ROIs outlining the basilar artery plaque on T1WI‐SPACE images of one patient. (c) Segmentations extracted from the ROIs corresponding to the images in part (b). ROI, region of interest; TOF, time of flight; T1WI SPACE, T1‐weighted imaging using sampling perfection with application‐optimized contrasts at various flip angles.

Plaque manual segmentation was performed by a physician with more than 5 years of diagnostic experience in HR‐VWI using Insight Segmentation and Registration Toolkit Snap (ITK‐SNAP, Version 3.8.0, http://www.itk-snap.org/). The level of basilar artery plaques of each patient on the plain‐scanning and enhanced HR‐VWI images was used for segmentation (Figure 1). Slight changes in patient positioning due to the longer interval between contrast injection and enhanced scanning and the small size of intracranial plaques necessitated separate outlining for plain and enhanced images.

Another radiologist with more than 5 years of diagnostic experience independently outlined ROIs for a randomized subset of patients (*n* = 40) from the study population to evaluate the reproducibility of ROI segmentation. Consistency between the two physicians′ ROI segmentations was assessed using the intraclass correlation coefficient (ICC), with an ICC > 0.8 indicating good consistency. When two physicians disagree, disagreements are resolved through consensus‐based discussion.

#### 2.5.2. Feature Extraction, Screening, and Model Construction

The original images were normalized using the *Z*‐score standardization method, adjusting the gray values to a standard normal distribution, before feature extraction. Radiomics features were extracted using the “PyRadiomics” package (https://www.radiomics.io/PyRadiomics.html) in Python. A total of 1316 radiomics features were extracted from the plain‐scanning and enhanced HR‐VWI images of each patient. To ensure result reliability, only features with an ICC > 0.8 from the two repeated measurements were retained (total of 875 features). Feature deredundancy was performed using the minimum redundancy maximum correlation method, whereas the LASSO algorithm was used to reduce the feature variables (this study employs the following standardized procedure: Firstly, the performance of different regularization parameters *λ* is evaluated within the training set via 10‐fold cross‐validation. Secondly, the optimal *λ* value is selected using the “1‐standard error [1‐SE] criterion,” whereby the model with the maximum regularization level [*λ*_1_se] within one standard error of the minimum cross‐validation error is chosen. This strategy prioritizes selecting simpler, more generalizable models while maintaining performance. Finally, the selected *λ*‐1se was used to refit the LASSO model across the entire training set to determine the final features. The entire process was rendered fully reproducible by setting a random seed, with all steps strictly isolated from the test set to prevent data leakage). The most suitable features were then identified through univariate and multivariate logistic regression analyses. These features were used to establish and validate three models: the plain‐scanning, enhanced, and plain − scanning + enhanced HR − VWI models. Finally, a combined model was constructed by integrating the selected clinical and traditional imaging features with the plain − scanning + enhanced HR − VWI radiomics features.

### 2.6. Statistical Analysis

Statistical analysis was performed using SPSS 26.0 (International Business Machines Corporation [IBM]). Normally distributed data were expressed as $\bar{x}\pm s$, and comparisons between groups were conducted using independent samples *t* tests. Categorical data were presented as frequencies and percentages, with comparisons between groups performed using the *χ* ^2^ test or Fisher′s exact test. The clinical and imaging characteristics were initially screened through univariate analysis, and variables with *p* < 0.05 were included in Firth‐penalized logistic regression analysis. The predictive efficacy of each model for symptomatic basilar atherosclerotic plaques was evaluated using receiver operating characteristic (ROC) curves and AUC. Nomograms were constructed to assess the performance of the combined model, whereas calibration curves and clinical decision curves were used to evaluate model reliability. The DeLong test was employed to compare the AUC values across different models. Differences were considered statistically significant at *p* < 0.05.

## 3. Results

### 3.1. Patients

Among the 154 patients, 107 were assigned to the training set (56 in the symptomatic group and 51 in the asymptomatic group) and 47 to the validation set (24 in the symptomatic group and 23 in the asymptomatic group). A total of 110 patients had a history of hypertension, 61 had diabetes, 87 had hyperlipidemia, 73 had a history of smoking, and 63 reported alcohol consumption. The two physicians demonstrated high agreement in ROI delineation (ICC > 0.8). No statistically significant differences (*p* > 0.05) were observed in the clinical or conventional imaging features between the training and validation sets.

### 3.2. Univariate and Multivariate Logistic Analyses

No statistically significant differences (*p* > 0.05) were observed in clinical characteristics such as sex, history of hypertension, diabetes mellitus, hyperlipidemia, smoking, or alcohol consumption between the symptomatic and asymptomatic groups. However, age demonstrated a statistically significant difference (*p* < 0.05) (Table 1).

**Table 1:** Comparison of clinical characteristics between symptomatic and asymptomatic groups.

Characteristic	Symptomatic group (*n* = 80)	Asymptomatic group (*n* = 74)	*p*
Sex			
Men	61	56	0.934
Women	19	18	
Age (year)	59.90 ± 9.145	63.09 ± 7.916	0.025
History of hypertension	54	56	0.263
History of diabetes	29	32	0.376
History of hyperlipidemia	44	43	0.698
Smoking history	37	36	0.766
History of alcohol consumption	33	30	0.929

For conventional imaging characteristics, statistically significant differences (*p* < 0.05) in IPH, *D*_v_, *S*_L_, enhanced plaque signal, narrowing rate, and plaque burden were observed between the two groups (Table 2).

**Table 2:** Comparison of conventional imaging features between symptomatic and asymptomatic groups.

Feature	Symptomatic group (*n* = 80)	Asymptomatic group (*n* = 74)	*p*
IPH (no. of patients)	49	23	0.000
*D*_p_ (mm)	3.923 ± 1.112	3.815 ± 1.061	0.537
*D*_v_ (mm)	0.739 ± 0.483	0.920 ± 0.518	0.028
Normal lumen diameter at the proximal end of the stenosis (mm)	3.397 ± 0.827	3.466 ± 0.792	0.597
*S*_L_ (cm^2^)	0.015 ± 0.013	0.021 ± 0.017	0.042
*S*_V_ (cm^2^)	0.213 ± 0.083	0.211 ± 0.105	0.915
Plain‐scanning plaque signal	400.213 ± 106.017	376.877 ± 118.549	0.201
Plain‐scanning peripheral gray matter signal	354.985 ± 65.866	351.625 ± 66.254	0.751
Enhanced plaque signal	756.410 ± 229.362	617.012 ± 233.465	0.001
Enhanced peripheral gray matter signal	358.763 ± 68.537	346.238 ± 71.060	0.267
Distal normal vessel area (cm^2^)	0.134 ± 0.051	0.133 ± 0.047	0.893
Narrowing rate	0.787 ± 0.124	0.736 ± 0.136	0.020
Plaque burden	0.930 ± 0.053	0.902 ± 0.074	0.010
Enhancement rate	0.910 ± 0.517	0.727 ± 0.635	0.054
Reshaping index	1.676 ± 0.625	1.623 ± 0.584	0.584

Abbreviations: *D*_p_, plaque diameter at stenosis; *D*_v_, vessel diameter at stenosis; IPH, intraplaque hemorrhage; *S*_L_, stenotic lumen area; *S*_V_, vessel area at stenosis.

**Figure 2:**
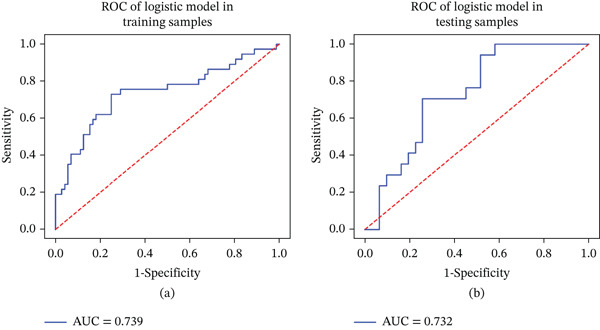
(a, b) The ROC of logistic model.

Incorporating statistically significant indicators into the Firth‐penalized logistic regression analysis confirmed that IPH and enhanced plaque signal were independent risk factors for predicting symptomatic basilar atherosclerotic plaques (Table 3). Collinearity analysis was performed before modeling for the two selected features, and no significant multicollinearity was detected. The logistic model yielded an AUC of 0.739 in the training sample (F1‐score: 0.642, precision: 0.591, recall: 0.703) and an AUC of 0.732 in the testing sample (F1‐score: 0.611, precision: 0.579, recall: 0.647) (Figure 2).

**Table 3:** Results of Firth‐penalized logistic regression analysis for predicting symptomatic basilar atherosclerotic plaque.

Variable	B (coefficient)	SE	*p*value	OR	95% CI
Age	−0.031	0.022	0.140	0.969	0.928–1.011
IPH	0.908	0.360	0.012	2.479	1.215–5.062
*D*_v_	0.275	1.102	0.802	1.316	0.15–11.523
*S*_L_	0.152	0.824	0.855	1.164	0.232–5.845
Enhanced plaque signal	0.002	0.001	0.040	1.002	1–1.004
Narrowing rate	0.423	1.876	0.821	1.527	0.038–61.82
Plaque burden	0.418	0.561	0.457	1.519	0.506–4.564

Abbreviations: CI, confidence interval; *D*_v_, vessel diameter at stenosis; IPH, intraplaque hemorrhage; OR, odds ratio; *S*_L_, stenotic lumen area.

### 3.3. Evaluation of the Effectiveness of Radiomics Model

**Figure 3:**
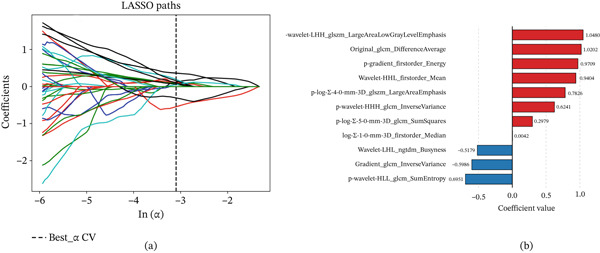
(a) The LASSO plot for feature selection. (b) The weight plot of the finally selected features.

Radiomics models were constructed using six features from plain‐scanning HR‐VWI (one first‐order energy feature and five texture features: the gray level co‐occurrence matrix (GLCM) features and two gray level size zone matrix (GLSZM) features) and five features from enhanced HR‐VWI (two first‐order statistical features and three texture features: two GLCM features and one Neighbouring Gray Tone Difference Matrix (NGTDM) feature). An additional combined model was built using 11 features derived from the plain‐scanning and enhanced HR‐VWI models (Figure 3).

**Figure 4:**
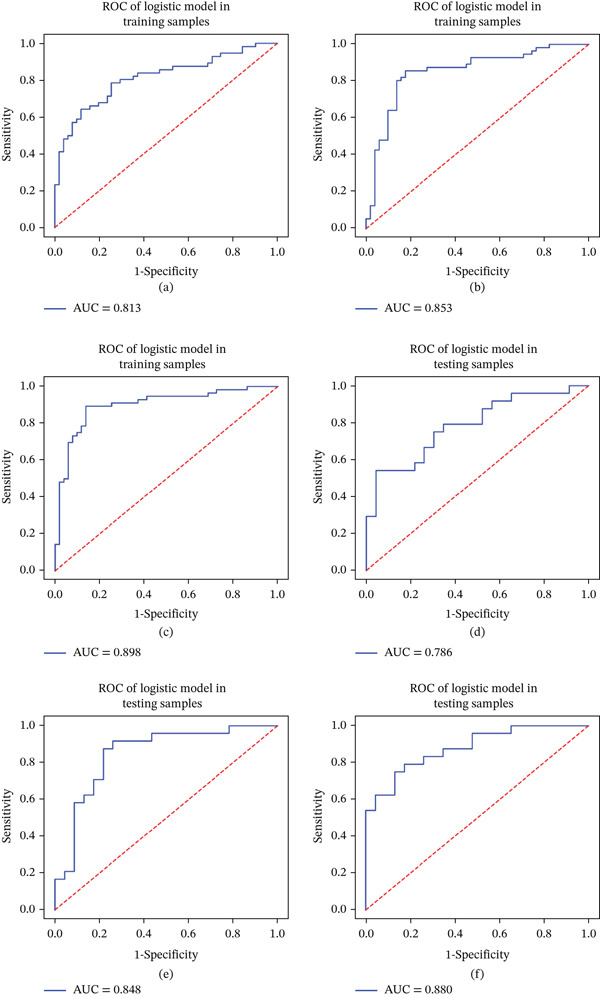
ROC curves representing the performance of the models for predicting symptomatic basilar atherosclerotic plaques: (a, b) plain‐scanning model for the training and validation sets, respectively; (c, d) enhanced model for the training and validation sets, respectively; (e, f) plain − scanning + enhanced model for the training and validation sets, respectively. In both the training and validation sets, the enhanced model outperformed the plain‐scanning model, whereas the plain − scanning + enhanced model demonstrated superior performance compared with the enhanced model. ROC, receiver operating characteristic.

In the training set, the AUC values for predicting symptomatic basilar atherosclerotic plaques were 0.813 (F1‐score: 0.768, precision: 0.768, recall: 0.768), 0.853 (F1‐score: 0.839, precision: 0.839, recall: 0.839), and 0.898(F1‐score: 0.875, precision: 0.875, recall: 0.875) for the plain‐scanning, enhanced, and plain − scanning + enhanced HR − VWI models, respectively (Figure 4a–c). In the validation set, the AUC values were 0.786 (F1‐score: 0.696, precision: 0.727, recall: 0.667), 0.848 (F1‐score: 0.824, precision: 0.778, recall: 0.875), and 0.880 (F1‐score: 0.776, precision: 0.760, recall: 0.792) for the plain‐scanning, enhanced, and plain − scanning + enhanced HR − VWI models, respectively (Figures 4d–f).

**Figure 5:**
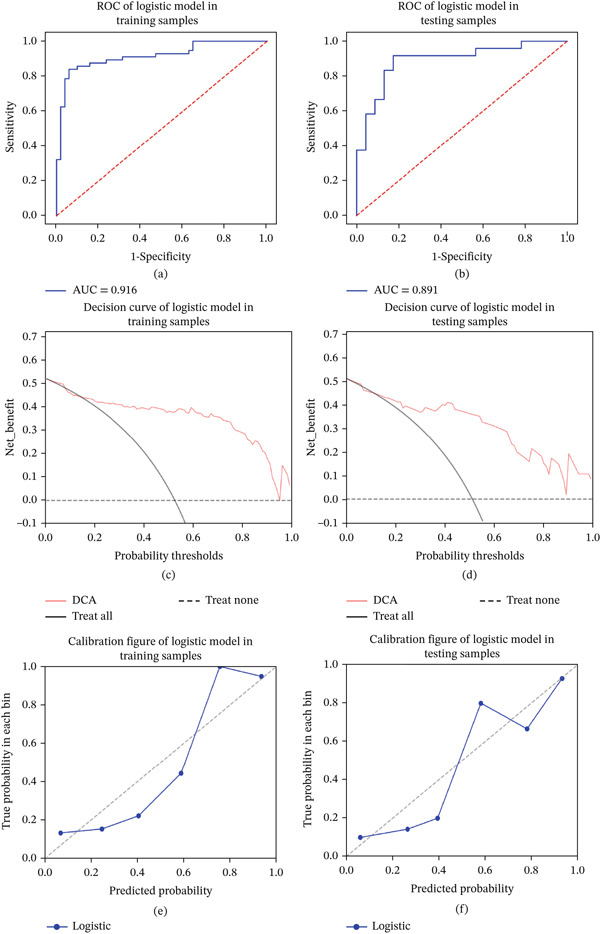
ROC curves for the combined model in (a) the training set and (b) the validation set. DCA curves for the combined model in (c) the training set and (d) the validation set. “Treat all” represents all predictions for symptomatic basilar artery plaques, and “Treat none” represents all predictions for asymptomatic plaques. The red line indicates the combined model. Calibration curves of the combined model in (e) the training set and (f) the validation set. The closer the blue line is to the diagonal, the more accurate the model′s predictions are. DCA, decision curve analysis; ROC, receiver operating characteristic.

**Figure 6:**
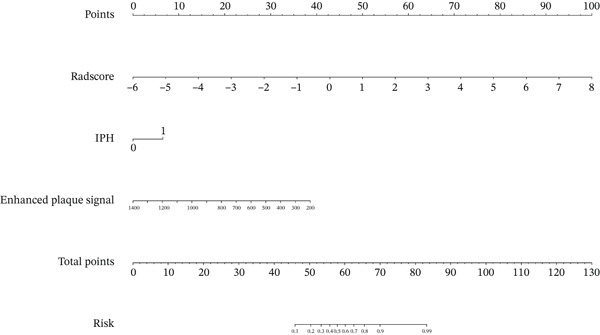
Nomograms for the combined model.

A combined model was established by combining IPH, enhanced plaque signal, and plain − scanning + enhanced HR − VWI radiomics features. In the training set, the combined model achieved an AUC value of 0.916 (F1‐score: 0.867, precision: 0.939, recall: 0.821) for predicting symptomatic basilar atherosclerotic plaques, with sensitivity, specificity, and accuracy of 0.821, 0.941, and 0.879, respectively. In the validation set, the AUC value of the combined model was 0.891 (F1‐score: 0.826, precision: 0.864, recall: 0.792), with sensitivity, specificity, and accuracy of 0.792, 0.870, and 0.830, respectively (Figure 5). Nomograms were constructed for the combined model (Figure 6), and calibration curves and clinical decision curves demonstrated good reliability and consistency of the combined model (Figure 5).

The results of the DeLong test showed statistically significant differences in the AUC values of the plain‐scan, enhanced, combined, and nomogram models compared with the traditional clinical imaging model (all *p* < 0.05). This suggests that the diagnostic efficacy of all these models is significantly superior to that of the traditional model (Table 4).

**Table 4:** Results of the DeLong test.

Group	*Z*value	*p*
Traditional model versus plain‐scan model	2.874	0.004
Traditional model versus enhanced model	3.215	0.001
Traditional model versus combined model	4.127	< 0.001
Traditional model versus nomogram model	3.891	< 0.001

## 4. Discussion

This study integrated clinical factors, conventional HR‐VWI imaging analysis, and radiomics features from plain‐scanning and enhanced HR‐VWI models to construct predictive models for identifying symptomatic basilar atherosclerotic plaques. The results indicated that among clinical and traditional HR‐VWI imaging features, IPH and enhanced plaque signals were independent predictors. The radiomic analysis accurately identified differences between symptomatic and asymptomatic basilar atherosclerotic plaques, with the enhanced HR‐VWI radiomics model outperforming the plain‐scanning model. The plain − scanning + enhanced HR − VWI radiomics model demonstrated superior performance compared with both plain‐scanning and enhanced models individually. The combined model, which combined IPH, enhanced plaque signals, and plain − scanning + enhanced HR − VWI radiomics features, was the most effective in predicting symptomatic plaques. These findings highlight the crucial role of radiomics analysis in improving the assessment of symptomatic basilar atherosclerotic plaques.

However, the diagnostic performance of the combined model was not considerably superior to the plain − scanning + enhanced HR − VWI radiomics model. This can be attributed to the higher weighting of the radiomics model, which demonstrated improved performance in the combined model compared with the traditional clinical and imaging features. This suggests that although traditional qualitative indicators are not entirely replaced, they may be overshadowed by the quantitative insights provided by radiomics features. It further emphasizes the potential for a more standardized and objective diagnostic process based on quantitative analysis.

Previous radiomics studies on atherosclerotic plaques have primarily focused on carotid plaques. Han et al. [11] in their nomogram based on T1‐weighted contrast‐enhanced (T1CE) demonstrated a strong predictive value for stroke risk assessment in carotid atherosclerotic plaques, achieving AUC values of 0.84 and 0.82 in the training and test cohorts, respectively. Similarly, Zhang et al. [12] used a radiomics approach to distinguish between symptomatic and asymptomatic carotid plaques, with their nomogram model achieving an AUC value of 0.986. However, these studies predominantly relied on 2D imaging and evaluated features only at the largest plaque level. In contrast, the present study employed 3D HR‐VWI to analyze imaging characteristics across the entire plaque. Carotid plaques allow for clearer visualization of internal components owing to their larger size, thereby emphasizing the benefits of multisequence scanning. In contrast, radiomics studies on intracranial atherosclerotic plaques, particularly those involving the basilar artery, remain relatively scarce.

Previous studies have primarily used HR‐VWI to explore the relationship between clinical imaging features and culprit plaques [13]. This study identified IPH as a predictor of symptomatic basilar atherosclerotic plaques. Schindler et al. [14] analyzed seven cohort studies and confirmed that IPH is an independent risk factor for clinical symptoms. IPH may occur through two mechanisms: First, plaque formation disrupts vascular endothelial cells, allowing erythrocytes to infiltrate the plaque and second, neovasculature forms within plaques to compensate for nutrient and blood supply during atherosclerosis. These vessels, being fragile and highly permeable, contribute to IPH [15]. Numerous studies have demonstrated that IPH incidence is higher in symptomatic patients than in asymptomatic patients [16–18], which was consistent with the findings of this study.

Additionally, this study demonstrated that the enhanced model considerably outperformed the plain‐scanning model in both the training and validation groups, highlighting plaque enhancement as another risk factor for ischemic stroke. Studies have demonstrated that plaque enhancement is strongly associated with ischemic stroke, with higher enhancement levels closely linked to culprit plaques and stroke [19]. Plaque enhancement is caused by neovascularization and increased endothelial permeability that facilitates the entry of contrast agents from blood plasma to accumulate within the plaque, leading to varying degrees of enhancement [20, 21].

Certain plaque features, such as IPH, are linked to an elevated risk of rupture and intracranial ischemic stroke. In contrast, metrics such as narrowing rate, plaque burden, and enhancement rate can predict future symptoms of basilar atherosclerotic plaques. However, these features are constrained by variability in observer expertise, limited reproducibility, and inconsistencies in imaging protocols. In contrast, radiomics—a computational approach that extracts and analyzes extensive quantitative features from medical images—provides objective quantitative data that is more comprehensive than traditional features and does not rely on radiologists′ subjective assessments. Through lesion segmentation and high‐throughput image feature extraction, it reveals vast amounts of information invisible to the naked eye. By surpassing human interpretation capabilities, it holds promise for enhancing diagnostic accuracy and confidence [22].

For instance, although IPH is recognized as a marker of high‐risk plaques, most prior studies have qualitatively identified it, lacking quantitative insights into signal intensity, volume, shape, or complex distribution patterns of IPH. The superior performance of radiomic features compared with traditional visual imaging indicators can be mechanistically explained by their ability to capture subtle, spatially resolved textural and intensity heterogeneity that is invisible to subjective human interpretation. First‐order statistical and energy features reflect global intensity distribution, whereas texture features derived from GLCM, GLSZM, and NGTDM quantify spatial correlations, regional size patterns, and local gray‐level differences within the plaque. These quantitative metrics correspond directly to microscale histopathological variations including focal IPH embedded in the LRNC, local thinning or discontinuity of the fibrous cap, heterogeneous inflammatory cell infiltration, and irregular calcification distribution. [23, 24] Such microstructural alterations are closely associated with plaque vulnerability but cannot be discriminated by conventional qualitative assessment. Therefore, radiomic features act as computational imaging surrogates of plaque microstructure and histology, enabling noninvasive identification of high‐risk characteristics that exceed the perceptual limits of visual interpretation. This explains why radiomics achieves improved performance in evaluating plaque composition and vulnerability compared with traditional imaging features. In our study, this limitation underscores why the combined model, integrating radiomics with clinical and traditional imaging features, surpassed conventional models, achieving a higher AUC and demonstrating superior performance in classifying high‐risk basilar artery plaques.

The variation in radiomics features extracted and selected from plain‐scanning and enhanced HR‐VWI images arises from the differing pathophysiologic characteristics of plaques reflected in T1‐weighted signals before and after contrast agent injection. For instance, a high signal on the plain T1‐weighted image indicates IPH. In contrast, a high signal on the enhanced T1‐weighted image likely reflects contrast agent uptake due to plaque neovascularization or active inflammation [25].

A recent study using MRI radiomics texture analysis [26] achieved an AUC value of 0.936 in distinguishing symptomatic basilar artery plaques, surpassing the AUC value of 0.833 obtained with clinical imaging features alone. Based on this evidence, we developed an HR‐VWI–based radiomics model for classifying basilar artery plaque. Unlike previous studies, our model incorporated both histogram and texture features, enhancing diagnostic accuracy. The first‐order features quantify the distribution of voxel values without considering spatial relationships and are derived through histogram analysis [27]. Second‐order, or “texture,” features characterize statistical relationships between voxels with varying contrast values [26]. Among the final 11 selected features in this study, only first‐order and texture features were consistently identified across both sequences, underscoring their value as key quantitative descriptors of plaques beyond visual radiologic assessment. First‐order statistical and energy features describe global gray‐level distribution, GLCM reflects local textural correlation, GLSZM quantifies connected regional structure, and NGTDM characterizes gray‐level difference and roughness. These five types of features complementarily describe plaque microstructural heterogeneity from different dimensions. High heterogeneity features, including high entropy, high contrast, high roughness, and high small zone proportion, correspond to vulnerable pathological changes such as inflammation, lipid accumulation, and cellular necrosis. In contrast, high uniformity, high correlation, and large zone structure indicate a stable phenotype dominated by fibrosis and calcification. This study confirms that multidimensional radiomic features enable noninvasive evaluation of plaque pathological components, providing an objective and quantitative imaging basis for clinical risk stratification [23, 24].

The evaluation of symptomatic intracranial atherosclerotic plaques provides critical value for risk stratification, therapeutic selection, and determination of optimal intervention timing in patients with ischemic stroke or TIA. Beyond luminal stenosis, plaque features including plaque enhancement, IPH, ulceration, and disrupted fibrous cap, as well as hemodynamic impairment and microembolic signals, serve as powerful predictors of recurrent stroke and help identify high‐risk individuals [28, 29]. Aggressive medical therapy remains the fundamental strategy for symptomatic intracranial atherosclerotic plaques. Endovascular treatment is not recommended as first‐line therapy but may be considered in highly selected patients with severe stenosis and recurrent ischemia despite optimal medical treatment or significant hemodynamic compromise. Regarding intervention timing, acute reperfusion therapy should be administered in eligible patients as early as possible. In the subacute stage, vessel wall imaging and perfusion evaluation help guide risk stratification and early intervention decisions. Long‐term imaging follow‐up is necessary to monitor plaque progression or regression in the chronic phase. In summary, comprehensive assessment of plaque characteristics and hemodynamic significance enables personalized management of symptomatic intracranial atherosclerotic disease, which is essential for optimizing clinical outcomes and reducing the risk of recurrent ischemic events.

This study had several limitations. First, the sample size was relatively small, particularly in the test cohort. A larger dataset is required to validate the accuracy of the predictive model. Second, the ROIs were manually segmented, which is especially challenging for small intracranial plaques. Automated segmentation could reduce interobserver variability and enhance the feasibility of applying this approach to larger datasets. Third, the conventional model included only two imaging features (IPH and enhanced plaque signal), as all imaging features associated with ischemic stroke may be too correlated with each other and thus they may be eliminated by multivariate analysis [30]. Fourth, all patients were enrolled from a specialized cardiovascular hospital, introducing inherent selection bias. This may explain the absence of significant between‐group differences in hypertension and diabetes, which could limit the generalizability of our results. Finally, this single‐center study lacked external validation, underscoring the need for future multicenter studies. Incorporating quantitative imaging data from large population‐based studies will facilitate the development of a reliable, automated HR‐VWI‐based stroke risk assessment tool.

## 5. Conclusions

HR‐VWI–based radiomics analysis demonstrated high accuracy in distinguishing symptomatic from asymptomatic basilar atherosclerotic plaques. In this study, the combined model integrating HR‐VWI imaging, clinical, and radiomics features proved most effective in identifying symptomatic plaques. This approach offered valuable insights for detecting high‐risk plaques linked to ischemic stroke. Further prospective studies are required to confirm the predictive potential of HR‐VWI radiomics analysis for future stroke risk in intracranial plaques.

## Author Contributions

Conceptualization: Juan Bai and Jiang Wu; methodology and software: Juan Bai, Ning Li, and Jingxi Yan; validation and formal analysis: Juan Bai and Ning Li; investigation: Juan Bai and Jingxi Yan; data curation: Xiaoyong Hao and Lina Zhu; writing—original draft: Juan Bai; writing—review and editing: Jiang Wu and Mengzhu Wang; visualization: Juan Bai and Ning Li; supervision: Jiang Wu and Caixian Yang; funding acquisition: Juan Bai. Juan Bai and Caixian Yang contributed equally to this study.

## Funding

This study was funded by the Scientific Research Incentive Fund of Shanxi Cardiovascular Hospital (Grant No. XYS20230201).

## Conflicts of Interest

The authors declare no conflicts of interest.

## Data Availability

Research data are not shared.
